# Factors Related to Oral Healthcare Service Utilization among Korean Adults Aged 25–79 Years

**DOI:** 10.3390/ijerph17176032

**Published:** 2020-08-19

**Authors:** Han-Na Kim, Sang-Jun Han, Eun-Joo Jun, Jin-Bom Kim

**Affiliations:** 1 Department of Dental Hygiene, College of Health and Medical Sciences, Cheongju University, Cheongju 28503, Korea; hnkim@cju.ac.kr; 2 Department of Preventive and Community Dentistry, School of Dentistry, Pusan National University, Yangsan 50612, Korea; drhan1215@gmail.com (S.-J.H.); jsilver@pusan.ac.kr (E.-J.J.); 3 Dental and Life Science Institute, Pusan National University, Yangsan 50612, Korea

**Keywords:** Andersen–Newman model, dental caries, education, health insurance, inequality, oral health care utilization, periodontitis, socioeconomic status

## Abstract

The factors related to oral healthcare service utilization (OHSU) among Korean adults aged 25–79 years were assessed using the Andersen model with the sixth Korean National Health and Nutrition Examination Survey data. The study included 12,937 participants aged 25–79 years who answered questions on the predisposing, enabling, and need factors related to OHSU at dental clinics within the past 1 year. Age, sex, and education level were selected as predisposing factors; household income, residence region, and national and private health insurance status as enabling factors; and self-perceived oral health, dental pain, chewing status, and discomfort while speaking as need factors. These factors were assessed using multivariable complex logistic regression models. OHSU at dental clinics within the past 1 year was lower among less-educated participants, those with low, middle–low, and middle–high household income levels, rural participants, those benefiting from the Medicaid system, and non-insured participants. OHSU was higher among older participants, those who rated their self-perceived oral health status as bad, those with experience of dental pain, and those who experienced discomfort while chewing and speaking. The need factors were the most influential. Thus, interventions to reduce inequalities in OHSU are required to promote oral health for all.

## 1. Introduction

Health is a fundamental factor in maintaining a good quality of life. Inequitable healthcare service utilization can result in health gaps among social classes. Elimination of the obstacles to equal medical service utilization can promote healthcare for all [1]. Inefficient delivery of healthcare services and the geographic and economic obstacles to greater accessibility are emerging as major challenges in providing equitable healthcare [2,3]. Similar to general healthcare service utilization, oral health inequality has increased because of differences in oral healthcare service utilization (OHSU) among social classes [4,5,6].

To establish the form and direction of national interventions to improve the usage of healthcare services, a thorough understanding of the current status of healthcare service utilization is essential. The Anderson model is a behavioral science model of healthcare service utilization that organizes and suggests the relationship between health and healthcare services in areas that do not appear to have commonalities, such as sociology, psychology, economics, and medicine [7]. It is often referred to as a predictive model because it is used to predict healthcare utilization [7,8]. The framework in this model predicts that a series of predisposing, enabling, and need factors influence the utilization of healthcare services by people. According to the model, predisposing factors include demographic and social structures. Enabling factors facilitate the individuals to use the services, usually involving factors such as income, access to free healthcare services, and availability and access to the services. Finally, need factors are those that motivate service use [9,10,11].

More specifically, predisposing factors refer to the characteristics of the individual and his/her family before the occurrence of the disease. They include age, sex, marital status, the number of family members, and education level. Enabling factors enable individuals to use healthcare resources and can be divided into family resources and health service resources. Family resources are related to the ability to pay for healthcare services, such as income and type of medical insurance, and healthcare service resources include the distribution of healthcare workers and institutions, transportation, and distance. Need factor refers to the level of injury and disease that motivates an individual to decide on the use of healthcare services as a method to recognize and cope with the disease, and can be classified into subjective and personal health assessment by a healthcare professional [8,11,12].

Gift [13] evaluated the Anderson model and found it to be useful for the analysis of OHSU. Subsequently, the Anderson model has been widely used in the analysis of OHSU among the elderly and vulnerable populations requiring healthcare [14,15]. Evashwick et al. [16] reported that the predisposing factors were more important than other factors in OHSU prediction. In Korea, a few studies have used the Anderson model for the analysis of OHSU in population groups, such as women, residents of small and medium-sized cities and rural areas, college students, and children [12,17,18]. Ahn et al. [19] reported factors related to OHSU among the elderly by using the 2010–2012 Korean National Health and Nutrition Examination Survey (KNHANES) data obtained by the Korea Center for Disease Control and Prevention (KCDC). Kim and Lee [11] reported the factors related to OHSU among adults over 20 years of age by using the “National Health and Health Consciousness Behavior Survey 1995”.

National health insurance program has three types, including employee insurance, self-employed insurance, and Medicaid. The employee insurance category includes the insured person’s spouse, descendants, brothers or sisters, and direct lineal ascendants. Insured employees pay 5.08% of their average salary in contribution payments. Contribution rates change every year. The self-employed insurance category includes people excluded from the category of insured employees. Their contribution amount is set considering their income, property, living standard, and rate of participation in economic activities. The remaining population is supported by the Medical Aid [20].

However, all citizens became eligible for national health insurance in 1989, and since 2012, prosthetic treatments for tooth loss, such as dentures were included within the national health insurance coverage for the elderly, causing rapid changes in OHSU [21]. Therefore, to increase equity in OHSU for the entire population, measures to eliminate obstacles to OHSU based on the latest data are essential. Thus, the purpose of this study was to analyze the factors related to OHSU among Koreans by using the Anderson model with the sixth KNHANES data of the adult population aged 25–79 years.

## 2. Materials and Methods

### 2.1. Study Design and Participants

Data from KNHANES VI conducted by the Division of Chronic Disease Surveillance, KCDC, were used in this study. The KNHANES is a population-based cross-sectional survey designed to assess the health-related behavior, health condition, and nutritional state of Koreans. It was conducted as a tri-annual survey for the first three cycles, implemented in 1998, 2001, and 2005. Beginning with the fourth cycle of 2007, it was converted to an annual survey. The KNHANES VI (2013–2015) data was recently opened to the public and is free for all researchers who want to use it [22]. In the KNHANES VI, the total number of participants surveyed was 29,321; 22,948 survey participants completed at least one of the health, screening, and nutrition surveys, and the participation rate was 8.3%. A total of 16,049 participants were aged 25–79 years; among these, 12,937 participants who completed all the survey questionnaires were selected as the study population.

The sampling protocol for the KNHANES was designed to provide a complex, stratified, multistage, and probability cluster survey of the representative sample of the non-institutionalized civilian population in Korea. A detailed description of the sampling methods and survey contents can be found in previous publications [23,24]. Trained interviewers conducted face-to-face interviews using a structured questionnaire. All participants gave written informed consent. The institutional review board of the KCDC approved the KNHANES (2013-07CON-03-4C, 2013-12EXP-03-5C, and 2015-01-02-6C) [25].

### 2.2. Variables

**Figure 1:**
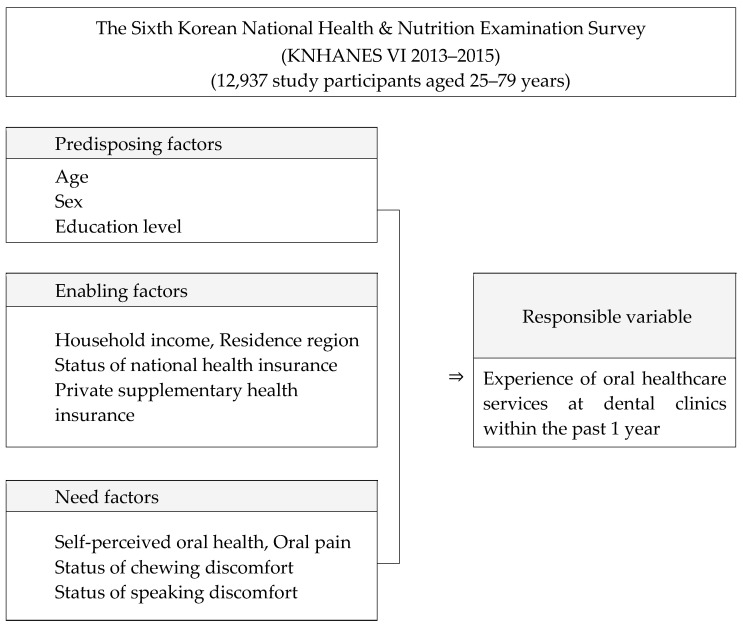
Variables of the study and the analysis process.

Among the factors surveyed through the interviews and surveys, the predisposing factors, enabling factors, and need factors related to oral healthcare services were selected for analysis. In the interview survey, dentists conducting oral examinations recorded answers directly from the participants in the survey. In the questionnaire survey, the participants’ self-reported answers to the questions were sought. Based on the Anderson model, variables corresponding to three areas were selected. Among the factors selected as independent variables, the predisposing factors were age, sex, and education level; the enabling factors were household income, residential area, medical insurance type (national health insurance, including employee, self-employed, and Medicaid benefits), and private supplementary health insurance; while the need factors were subjective oral health status, toothache, mastication discomfort, and pronunciation discomfort. The dependent variable was utilization of dental clinics in the past 1 year (Figure 1).

Subjective oral health was assessed using the question “When you think about yourself, what do you think of your oral health, such as teeth and gums?” Toothache was assessed using the question “Have you experienced toothache in the past 1 year?” Toothache was described as sore or throbbing teeth, sore, cold, or hot sensation, or discomfort when drinking or eating food. In response, the participants could choose “I have” or “I don’t have”. Discomfort was assessed by the question “Do you feel uncomfortable when chewing food due to problems in your mouth, such as teeth, dentures, and gums?” Pronunciation discomfort was assessed with the question “Are you currently uncomfortable with pronouncing words clearly because of problems in your mouth, such as teeth, dentures, and gums?” The response to the question “whether or not you have visited a dental clinic in the past 1 year” was used as the dependent variable.

Among the predisposing factors, the level of education was categorized as “Elementary school graduate”, “Junior high school graduate”, “High school graduate”, and “College graduate”. Among the enabling factors, household income was categorized as “Low”, “Middle–low”, “Middle–high”, and “High”. Residence region was classified as “Rural” for those living in the rural areas and “Urban” for the residents of towns or cities. The national health insurance status was categorized as “Employee”, “Self-employed”, and “Medicaid”. Among the need factors, the response to the question on subjective oral health status was classified under the “Positive recognition group (Fair)” if the reply was “Very good”, “Good”, or “Normal” and under the “Negative recognition group (Bad)” if the reply was “Bad” and “Very bad”. The response to the question assessing chewing discomfort was classified under “Yes” if the reply was “very uncomfortable” or “uncomfortable” and under “No” if the reply was “just so”, “not uncomfortable”, or “not at all uncomfortable”. A similar approach was employed for classifying the response to the question on discomfort while speaking. As for the dependent variable, participants who answered “Yes” to the question asking whether they had visited a dental clinic in the past 1 year were classified as those who had experienced OHSU.

### 2.3. Statistical Analyses

Statistical analyses were performed using IBM SPSS 25.0^®^ (IBM Corp., New York, NY, USA). The complex sampling design of the survey was considered to obtain the variances, and the individual weighted factors were used. A complex-sample multivariable logistic regression analysis with weights applied was performed to analyze the relationship between related variables and the OHSU after adjusting for potential confounders. After calculating the distribution ratio of each variable in each factor for the entire group of participants aged 25–79 years, three predictive models were devised. The results of the analysis were expressed as odds ratios (ORs) and 95% confidence intervals (CIs), and the significance levels of differences between groups was judged to show a type I error of 0.05.

## 3. Results

Table 1 shows the characteristics of the participants. The study contained more women than men, more college graduates than individuals with lower education levels, and more participants with a high-income level than those in the lower household income quartiles. A greater number of participants lived in urban areas, and the largest number of individuals with health insurance were the employees enrolled in national health insurance. Among these participants, those with private supplementary health insurance outnumbered those without. With respect to subjective oral health perception, the positive perception group contained more participants than the negative group, and there were more participants with no toothache experience and no discomfort while chewing or speaking (Table 1).

Three predictive models for the detailed factors affecting the OHSU were prepared. In model 1, the factors influencing the experience of OHSU were age and education level, although sex was not confirmed.

In model 2, the factors influencing the experience of OHSU were age, education level, household income, health insurance type (national health insurance and Medicaid), and private health insurance. In this model, the rate of experience of OHSU increased with age, and the lower the education level, the lower the household income. OHSU experience was less in those covered under Medicaid than in those covered by workplace health insurance and less in those who did not have private health insurance.

The factors affecting the experience of OHSU in model 3 were age, education level, household income, residence region, and private supplementary health insurance. The need factors that were significantly confirmed to affect utilization were self-perceived oral health status, dental pain, chewing discomfort, and discomfort while speaking.

Among the predisposing, enabling, and need factors, dental pain (adjusted OR = 2.53, 95% CI = 2.28–2.80) was the most influential factor with the highest OR for the experience of the OSHU (Table 2).

Table 3 shows the prediction model for specific factors affecting toothache experience. In the crude logistic regression analysis for dental pain, the level of education, household income, residence region, health insurance type, age, and sex were significantly confirmed as affecting variables, although private supplementary health insurance coverage was not relevant. In the adjusted model of dental pain, factors influencing toothache experience were education level, residential area, and health insurance type. The most influential factor influencing the experience of dental pain was the type of health insurance, and dental pain was estimated to be 1.36 times more likely to be experienced by the participants eligible for Medicaid benefits compared with the insured employees (Table 3).

## 4. Discussion

This study used the sixth 2013–2015 KNHANES data to devise a plan to improve oral health equity in Korea and used the Anderson model to determine the factors affecting the OHSU. The prediction models identified different significant variables. The reason why the KNHANES has more women than men in the number of participants could be that men who are busy at work may not have been able to respond to questionnaires and surveys because oral examinations, full body examinations, and long-term interviews were conducted on an empty stomach early in the morning. However, through the consultation of the Korea Population and Housing census conducted by the National Statistical Office, samples were selected according to the sex and age ratio of the region to represent the nation. Therefore, its value is sufficiently recognized as a material representing the nation.

Among the models that analyzed the factors affecting the experience of the OHSU, prediction model 1, which was composed of the predisposing factors, showed significant differences only in age and education level. Prediction model 2 showed significant differences in age, education level, household income, type of health insurance, and private supplementary health insurance. Differences based on the type of health insurance showed increased experience of OHSU among the participants covered under the Medicaid system compared with the insured employees. These results indicate that Medicaid coverage greatly improved access to dental clinics even in the lower economic class. It could be inferred that people covered under the Medicaid system visited dental clinics more for curative treatments because their oral health was worse than that of the insured employees. However, there may be a criticism that dental treatments may be easily provided to the people covered under the Medicaid system because of no economic burden of clinical treatments and, therefore, further study is warranted. National health insurance is a system in which all citizens except those from the low socioeconomic strata enroll compulsorily, pay insurance premiums, and receive benefits according to their income. In contrast, citizens from low-socioeconomic groups receive benefits from the Medicaid system, which provides all treatments from the national budget without the requirement of paying any premium. National health insurance does not cover some treatments, such as cosmetic plastic surgery and prosthetic treatments for lost teeth under 65 years of age. Those treatments not covered by national health insurance are totally paid by subscribers themselves. However, even for the medical items covered by the national health insurance, the subscribers must pay 10–60% of the medical expenses, depending on the type of treatment and the level of the treatment institution. For this reason, private supplementary health insurance is offered by private insurance companies. This insurance is not compulsory, although individuals may choose this insurance and pay premiums accordingly. This insurance pays for the expenses of medical items that are not covered by the national health insurance.

In predictive model 3, factors affecting the experience of OSHU were age, education level, household income, residence region, private supplementary health insurance coverage, self-perceived oral health, toothache experience, discomfort while chewing, and discomfort while speaking. The residence region, which was not significant for OHSU in prediction model 2, was a significant factor in prediction model 3, which included all the predisposing, enabling, and need factors. Thus, the dental visit experience of participants in the rural areas was lower than that of those living in the cities because dental clinics are concentrated in the cities and rarely distributed in the rural areas, resulting in poor geographical access [26,27].

Significant variables were also identified differently according to the prediction model. Prediction model 2 identified the type of health insurance as a significant factor, although this factor was considered non-significant in prediction model 3. This result may indicate that economic obstacles to accessing dental clinics have been alleviated because of the complete implementation of national health insurance and Medicaid benefits and the expansion of oral healthcare [28]. However, the type of health insurance, a significant factor in prediction model 1, was not significant in prediction model 3. This could have occurred because model 3 was doubly adjusted by adjusting the need factors in relation to the predisposing and enabling factors. For OHSU, the predisposing factors and enabling factors are demographic and socioeconomic factors. Socioeconomic factors can affect oral health status and consequently increase the need factors [5,6,29,30,31,32].

Participants with dental pain and discomfort while chewing and speaking, i.e., the specific need factors, had high OHSU experience. These results indicate that OHSU in Korea is still focused on post-disease curative treatments and prosthetic treatment after tooth loss.

Overall, the most influential factor in OHSU was dental pain. The second most influential factor was educational level. The OR of OHSU for participants with dental pain was 2.53, suggesting that people with dental pain were 2.53 times more likely to visit dental clinics than those without dental pain. Moreover, compared with those with college education, the OR of OHSU among primary school graduates was 0.59, indicating that OHSU among primary school graduates was only 58.9% of that among college graduates. Among the specific factors affecting dental pain, the most influential factor was the type of health insurance. The analysis results estimated that those with Medicaid benefits were 1.36 times more likely to experience dental pain compared with the insured employees. This suggests that participants with Medicaid benefits still tended to postpone treatment until they felt pain. Therefore, because participants with Medicaid benefits still appear to be facing difficulties in OSHU, a policy to resolve this inequity may be necessary.

Strayer et al. [33] reported that when the Anderson model was applied to US veterans’ OHSU, the need factors had the highest impact, and the enabling factors had the lowest impact. This is similar to the results of this study, which was conducted among Koreans.

In a previous study that analyzed OHSU among Korean women using the Anderson model, the inactivity because of oral diseases, a need factor, and the number of days of active constraints were the most important factors for OHSU, and the economic burden of treatment expense also had a significant impact [34]. These findings indicated that socioeconomic factors had a major influence on OHSU. Lee and Kim [34] reported that need factors were the most explanatory factors for oral treatment utilization among college students. Ahn et al. [19] analyzed OHSU among the elderly using the KNHANES 2010–2012 data and reported that the experience of OHSU increased 1.34 times when chewing discomfort occurred.

Meanwhile, in the 2017 Korea Community Health Survey data, the most important reasons for the lack of OSHU were stated as: “because I could not leave school or work”, followed by “economic reasons” [33]. Kim and Lee [34] analyzed the factors affecting unmet dental needs according to the oral health status of adults using the sixth KNHANES data and reported that economic reasons, such as household income, accounted for a large proportion. In 2017, the national health insurance coverage rate was 60.3% in clinics and 63.8% in general hospitals but only 31.7% in dental clinics and 18.9% in dental hospitals [35]. These findings indicate an urgent need to expand the national health insurance coverage rate, since economic obstacles still play a major role in OHSU.

In conclusion, the factors influencing the experience of OHSU among Korean people were education level among the predisposing factors; household income, type of health insurance, and private supplementary health insurance among the enabling factors; and dental pain among the need factors. The specific factors constituting the need factors had the greatest influence on the experience of OHSU.

This study was a cross-sectional study using the KNHANES VI data and analyzed only some of the factors related to the experience of OHSU. However, the number and the types of oral healthcare services could not be analyzed, which was one of the limitations of the study, the significance of the findings is that it allowed analysis of the factors related to OHSU using the national survey data that can represent Korean population. A comprehensive future study analyzing demographic and socioeconomic factors along with psychological factors and oral health status in relation to OHSU could help establish measures to alleviate the gap in OHSU because of social class. Moreover, the prevalence of oral diseases, such as dental caries and periodontitis is high in the socioeconomically underprivileged population and countries [36]. Active development of these preventive strategies can alleviate the oral health inequities experienced by underprivileged populations and countries.

## 5. Conclusions

This study analyzed the factors related to the experience of OHSU among Korean adults by using the Anderson model with the sixth KNHANES data to eliminate obstacles to the OHSU among Korean adults and increase equity. Among the predisposing, enabling, and need factors, the specific factors constituting the need factors had the greatest influence on OHSU. The factors influencing the experience of OHSU among Korean people were as follows: education level among the predisposing factors; household income, type of health insurance, and private supplementary health insurance among the enabling factors; and dental pain among the need factors. The most influential factor for dental pain was the type of health insurance, and the probability of OHSU for dental pain through Medicaid benefits was estimated to be 1.36 times than the employee insurance. Further studies are warranted to identify the diverse factors related to OHSU to alleviate barriers and improve oral health equity.

## Figures and Tables

**Table 1:** Distribution of study participants by variables.

Variables	Contents	Frequency (N)	Weighted %	*p*-Value *
Sex	Man	5493	48.9	0.010
	Woman	7444	51.1	
Education	Primary	2904	17.0	<0.001
	Middle	1432	10.0	
	High	3917	33.7	
	College	4244	39.3	
Household income	Low	2296	13.9	<0.001
	Middle–low	3246	24.2	
	Middle–high	3571	30.1	
	High	3768	31.7	
Region	Rural	2530	17.9	<0.001
	Urban	10,407	82.1	
Health insurance	Medicaid	436	2.9	
	Self-employed	4184	32.8	
	Employee	8317	64.3	
Private insurance	No	3084	19.5	<0.001
	Yes	9853	80.5	
Self-perceived oral health	≤Bad	5800	44.1	<0.001
	≥Fair	7137	55.9	
Dental pain	No	4950	38.6	<0.001
	Yes	7987	61.4	
Chewing discomfort	No	3159	21.1	<0.001
	Yes	9778	78.9	
Speaking discomfort	No	1345	8.5	<0.001
	Yes	11,592	91.5	

* Complex sample chi-square test.

**Table 2:** Impact of the predisposing, enabling, and need factors related to oral healthcare service utilization.

Categories	Variables	Contents	Adjusted OR (95% CI)		
			Model 1	Model 2	Model 3
Predisposing factors	Age		1.02 (1.02–1.02)	1.02 (1.02–1.03)	1.02 (1.02–1.03)
	Sex (Ref. = Woman)	Male	0.94 (0.87–1.01)	0.96 (0.89–1.03)	0.95 (0.87–1.03)
	Education level (Ref. ≥ College)	Primary	0.47 (0.40–0.54)	0.64 (0.55–0.73)	0.59 (0.51–0.68)
		Middle	0.65 (0.56–0.76)	0.79 (0.68–0.92)	0.73 (0.62–0.86)
		High	0.75 (0.68–0.84)	0.80 (0.72–0.89)	0.81 (0.72–0.90)
Enabling factors	Household income (Ref. = High)	Low		0.66 (0.56–0.78)	0.62 (0.53–0.73)
		Middle–low		0.74 (0.66–0.83)	0.72 (0.64–0.81)
		Middle–high		0.84 (0.75–0.93)	0.82 (0.74–0.92)
	Residence region (Ref. = Urban)	Rural		0.92 (0.81–1.04)	0.86 (0.76–0.99)
	National health insurance (Ref. = Employee)	Medicaid		1.33 (1.02–1.72)	1.25 (0.96–1.62)
		Self-employed		0.99 (0.90–1.09)	0.98 (0.89–1.08)
	Private insurance (Ref. = Yes)	No		0.84 (0.74–0.95)	0.83 (0.73–0.94)
Need-based factors	Self-perceived oral health (Ref. ≥ fair)	≤Bad			0.80 (0.73–0.87)
	Dental pain (Ref. = None)	Yes			2.53 (2.28–2.80)
	Chewing discomfort (Ref. = None)	Yes			1.17 (1.03–1.33)
	Speaking discomfort (Ref. = None)	Yes			1.26 (1.06–1.48)

Dependent variable: experience of oral healthcare services at a dental clinic within the past 1 year (reference category = No); adjusted odds ratio (OR) (95% confidence interval (CI)): adjusted odds ratio (95% confidence interval); model 1 was adjusted for age, sex, and education level; model 2 was adjusted for age, sex, education level, household income, region, and national health insurance and private insurance statuses; model 3 was adjusted for age, sex, education level, household income, region, national health insurance and private insurance statuses, self-perceived oral health, oral pain, chewing discomfort, and speaking discomfort.

**Table 3:** Impact of predisposing and enabling factors on dental pain experience.

Categories	Variables	Contents	Crude OR (95% CI)	Adjusted OR (95% CI)
Predisposing factors	Age		1.00 (1.00–1.00)	1.00 (0.99–1.00)
	Sex (Ref. = Woman)	Male	1.05 (0.97–1.14)	1.06 (0.97–1.15)
	Education level (Ref. ≥ College)	Primary	1.17 (1.03–1.33)	1.17 (0.99–1.38)
		Middle	1.29 (1.11–1.50)	1.29 (1.09–1.52)
		High	0.98 (0.89–1.09)	0.97 (0.87–1.08)
Enabling factors	Household income (Ref. = High)	Low	1.29 (1.12–1.48)	1.16 (0.99–1.35)
		Middle–low	1.12 (0.99–1.26)	1.04 (0.92–1.17)
		Middle–high	1.11 (0.99–1.25)	1.07 (0.95–1.20)
	Residence region (Ref. = Urban)	Rural	1.32 (1.15–1.52)	1.30 (1.13–1.50)
	National health insurance (Ref. = Employee)	Medicaid	1.46 (1.16–1.83)	1.36 (1.07–1.74)
		Self-employed	1.05 (0.96–1.15)	1.06 (0.96–1.16)
	Private insurance (Ref = Yes)	No	1.08 (0.97–1.19)	0.98 (0.87–1.10)

Dependent variable: dental pain experience within the past 1 year (reference category = No); adjusted OR (95% CI): adjusted odds ratio (95% confidence interval); model was adjusted for age, sex, education level, household income, region, national health insurance, and private insurance.
